# Co-crystal sustained by π-type halogen-bonding inter­actions between 1,4-di­iodo­perchloro­benzene and naphthalene

**DOI:** 10.1107/S2056989023008356

**Published:** 2023-09-29

**Authors:** Eric Bosch, Eric W. Reinheimer, Daniel K. Unruh, Ryan H. Groeneman

**Affiliations:** a Department of Chemistry and Biochemistry, Missouri State University, Springfield, MO 65897, USA; b Rigaku Americas Corporation, The Woodlands, TX 77381, USA; c Office of the Vice President for Research, University of Iowa, Iowa City, IA 52242, USA; d Department of Natural Sciences and Mathematics, Webster University, St. Louis, MO 63119, USA; Texas A & M University, USA

**Keywords:** halogen bonding, co-crystal, Type I chlorine-chlorine contacts, Type II iodine-chlorine contacts

## Abstract

The formation and crystal structure of a co-crystal based upon 1,4-di­iodo­perchloro­benzene (C_6_I_2_Cl_4_) as the halogen-bond donor along with naphthalene (nap) as the acceptor is reported. The co-crystal [systematic name: 1,2,4,5-tetra­chloro-3,6-di­iodo­benzene–naphthalene, (C_6_I_2_Cl_4_)·(nap)] generates a chevron-like structure that is held together primarily by π-type halogen bonds (*i.e*. C—I⋯π contacts) between the components. In addition, C_6_I_2_Cl_4_ also inter­acts with the acceptor *via* C—Cl⋯π contacts that help stabilize the co-crystal. Within the solid, both aromatic components are found to engage in offset and homogeneous face-to-face π–π stacking inter­actions. Lastly, the halogen-bond donor C_6_I_2_Cl_4_ is found to engage with neighboring donors by both Type I chlorine–chlorine and Type II iodine–chlorine contacts, which generates an extended structure.

## Abstract 2

The formation of a co-crystal sustained by π-type halogen bonds involving 1,4-di­iodo­perchloro­benzene and naphthalene is reported.

## 1. Chemical context

Halogen bonding continues to be a highly utilized non-covalent inter­action in the formation of multicomponent mol­ecular solids such as co-crystals. Halogen bonding is an attractive inter­action between an electrophilic region on a halogen atom and a nucleophilic region on a second atom (Gilday *et al.*, 2015 ▸). This electrophilic or positive region, namely the σ-hole, is located at the tip of a halogen atom bound to a carbon that inter­acts with a lone pair on an atom or an electron-rich aromatic surface (Cavallo *et al.*, 2016 ▸). In general, iodine generates the largest positive σ-hole when combined with neighboring electronegative atoms such as fluorine. The majority of these reported halogen bonds are classified as *n*-type meaning that the halogen atom is inter­acting with a lone pair such as on an N or O atom (Walsh *et al.*, 2001 ▸). A lesser investigated class of halogen bonds are π-type (*i.e*. C—I⋯π contacts) where the halogen atom inter­acts with an electron-rich surface such as a polycyclic aromatic hydro­carbon (Vainauskas *et al.*, 2020 ▸; d’Agostino *et al.*, 2015 ▸; Shen *et al.*, 2012 ▸). 
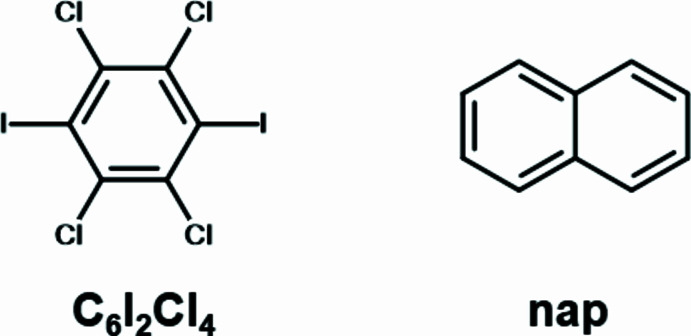


A continued goal within our research groups has been in the design and formation of halogen-bonded co-crystals based upon 1,4-di­iodo­perchloro­benzene (C_6_I_2_Cl_4_) as the donor. Recently, we reported the formation of photoreactive co-crystals based upon C_6_I_2_Cl_4_ along with *trans*-1,2-bis­(pyridine-4-yl)ethyl­ene (Bosch *et al.*, 2019*b* ▸) and 4-stilbazole (Bosch *et al.*, 2019*c* ▸) that are held together by primarily C—I⋯N or *n*-type halogen bonds. With the goal of expanding the type of halogen bonds that C_6_I_2_Cl_4_ can form in mol­ecular co-crystals, a study with a polycyclic aromatic was undertaken. Herein, we report the solid-state crystal structure of a co-crystal held together primarily by π-type halogen bonds between C_6_I_2_Cl_4_ and naphthalene (nap) resulting in a chevron-like structure. In addition to the π-type halogen bond, the co-crystal (C_6_I_2_Cl_4_)·(nap) is also held together by the combination of C—Cl⋯π contacts, homogeneous face-to-face π–π stacking inter­actions, Type I chlorine–chlorine contacts, and Type II iodine–chlorine contacts.

## 2. Structural commentary

**Figure 1:**
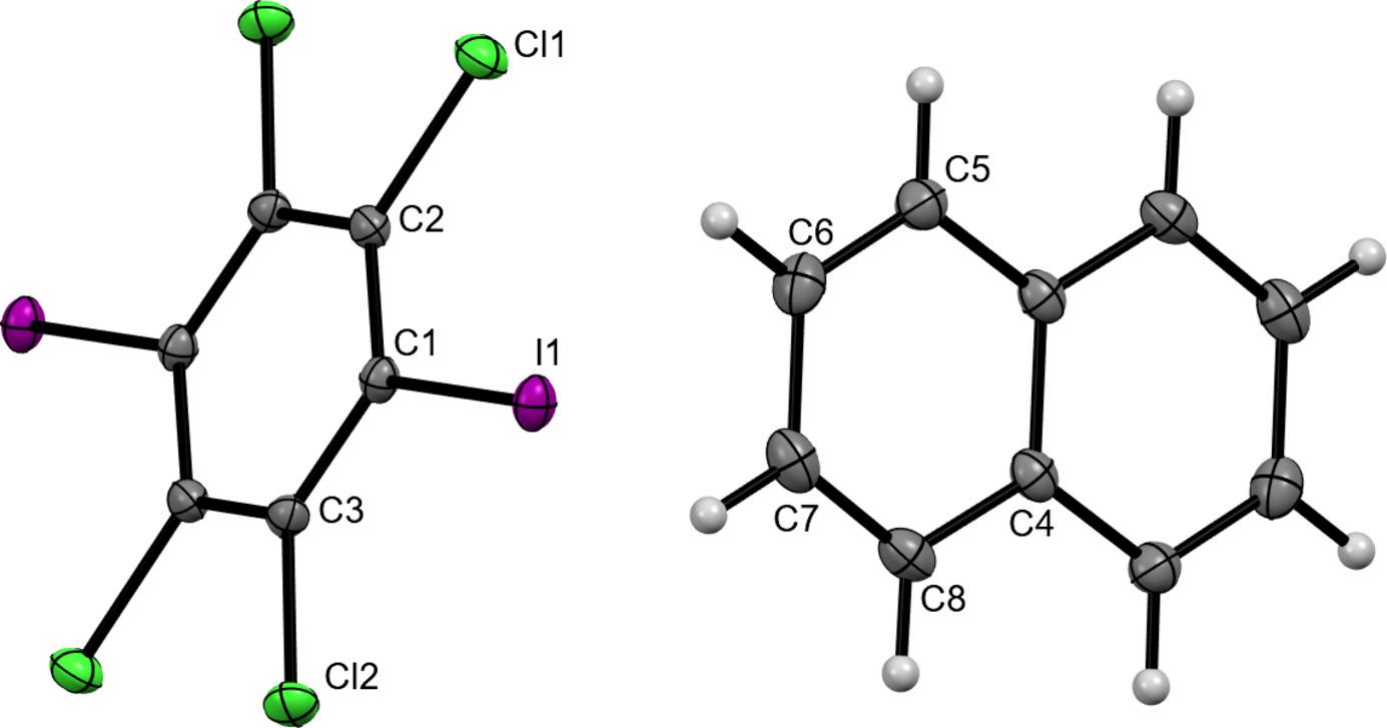
The labeled asymmetric unit of (C_6_I_2_Cl_4_)·(nap). Displacement ellipsoids are drawn at the 50% probability level for non-hydrogen atoms while hydrogen atoms are shown as spheres of arbitrary size.

**Figure 2:**
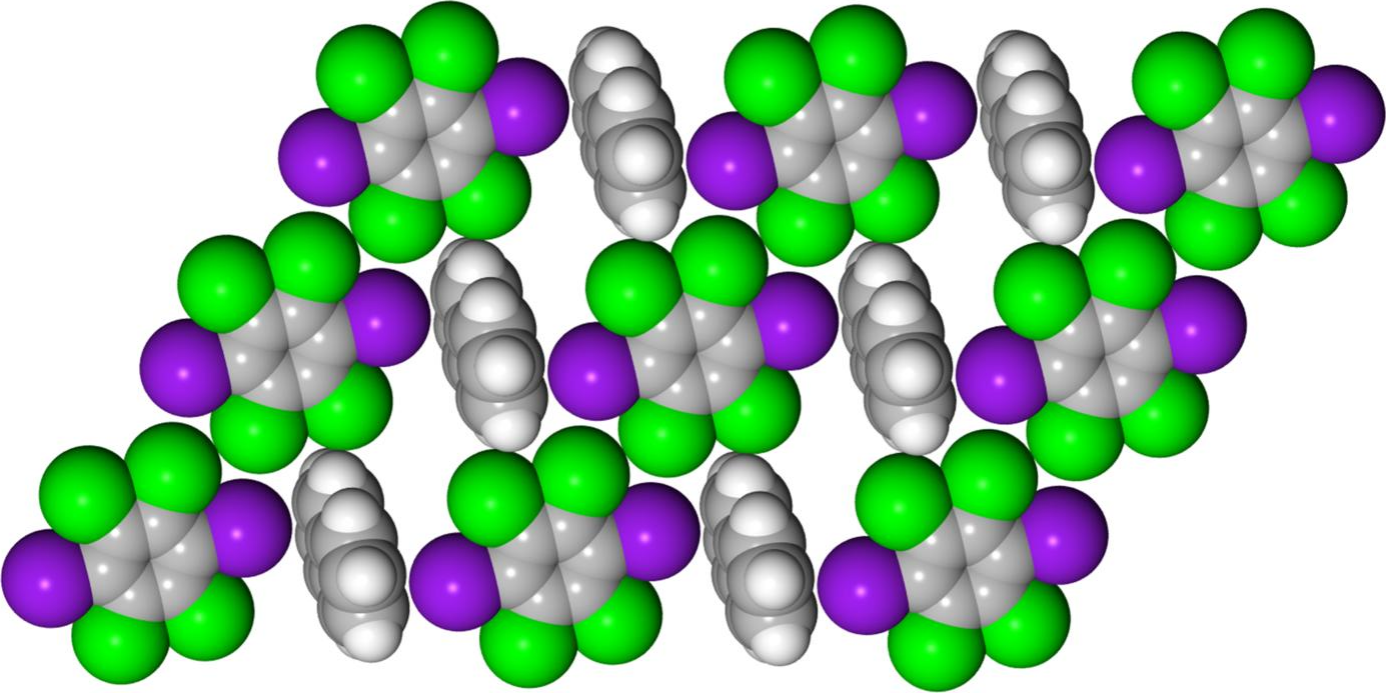
X-ray crystal structure of (C_6_I_2_Cl_4_)·(nap) illustrating the chevron-like packing pattern along with π-type halogen bonds. In addition, the Type I chlorine–chlorine and Type II iodine–chlorine inter­actions between neighboring chevron-based chains are also shown.

Crystallographic analysis reveals that (C_6_I_2_Cl_4_)·(nap) crystallizes in the centrosymmetric triclinic space group *P*ī. The asymmetric unit contains half a mol­ecule of both C_6_I_2_Cl_4_ and nap where inversion symmetry generates the remainder of each mol­ecule (Fig. 1 ▸). The co-crystal is sustained by π-type or C—I⋯π halogen bonds with a distance of 3.373 (1) Å along with a nearly perpendicular halogen-bond angle of 90.99 (4)° (Fig. 2 ▸). This halogen-bond distance and angle were determined by using the I atom on C_6_I_2_Cl_4_ and the calculated plane for the nap mol­ecule. As expected, C_6_I_2_Cl_4_ forms two π-type halogen bonds with two different nap mol­ecules, generating a chevron-like pattern (Fig. 2 ▸).

## 3. Supra­molecular features

**Figure 3:**
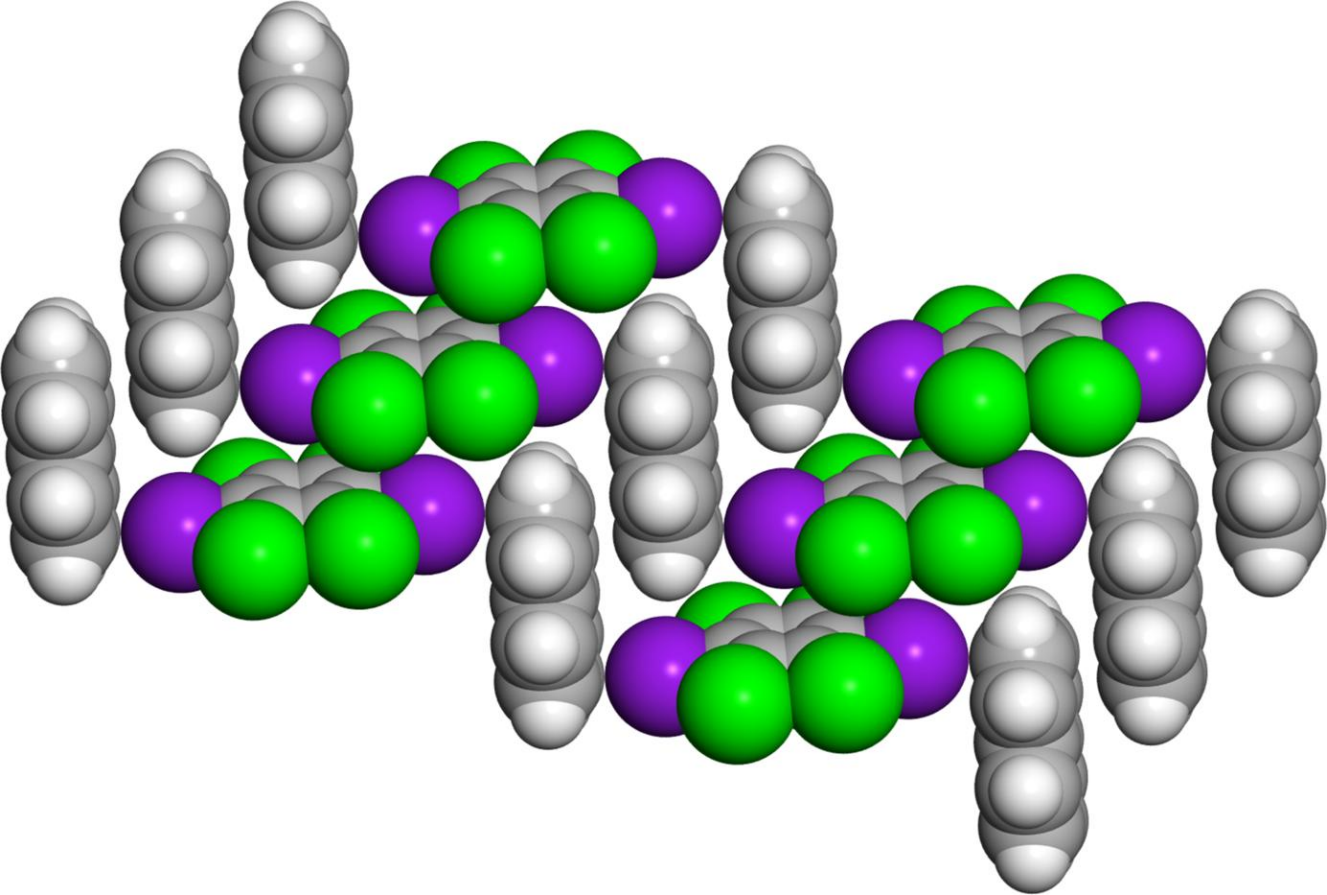
X-ray crystal structure of (C_6_I_2_Cl_4_)·(nap) illustrating the π-type halogen bonds and the offset face-to-face stacking of both the halogen-bond donor and acceptor.

In addition to π-type halogen bond within (C_6_I_2_Cl_4_)·(nap), the donor C_6_I_2_Cl_4_ is found to engage in Type I chlorine–chlorine contacts (Fig. 3 ▸). These inter­actions are found between crystallographically equivalent Cl atoms, namely Cl2⋯Cl2^i^ [symmetry code: (i) 1 − *x*, -*y, 1* − *z*], with a distance of 3.499 (1) Å and a C—Cl⋯Cl bond angle of θ_1_ = θ_2_ = 132.16 (6)° (Mukherjee *et al.*, 2014 ▸; Desiraju & Parthasarathy, 1989 ▸). In addition, neighboring donors also inter­act *via* Type II iodine–chlorine contacts. This inter­action is found between I1⋯Cl2^i^ [symmetry code: (i) 1 − *x*, -*y, 1* − *z*], with a distance of 3.808 (1) Å and a C—I⋯Cl bond angle of 111.83 (4)°. Both the aromatic halogen-bond donor and acceptor are found to engage in an offset and homogeneous face-to-face π–π stacking arrangement that stabilizes the co-crystal (Fig. 3 ▸). Lastly, C_6_I_2_Cl_4_ is inter­acting with two additional nap mol­ecules *via* C—Cl⋯π contacts at a distance of 3.391 (2) Å measured for Cl1⋯C5.

**Figure 4:**
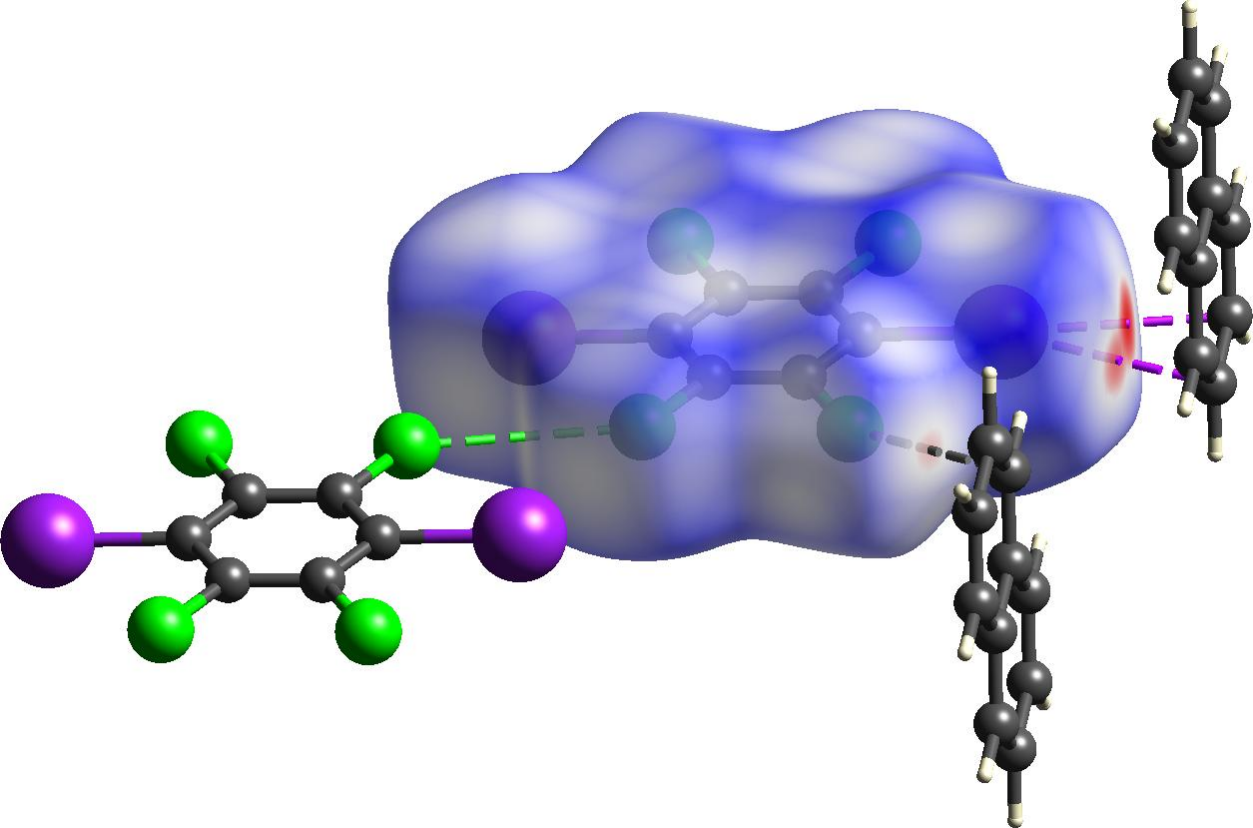
Hirshfeld surface of (C_6_I_2_Cl_4_)·(nap) where *d*_norm_ is mapped onto the surface illustrating the π-type halogen bonds (darkest red spots) and C—Cl⋯π contacts (faint red spots). Lastly, the Type I chlorine–chlorine inter­actions are shown with green dashed lines.

These various non-covalent inter­actions were also investigated and visualized by utilizing a Hirshfeld surface analysis (Spackman *et al.*, 2021 ▸) where *d*_norm_ is mapped onto the calculated surface (Fig. 4 ▸). The darkest red spots on the Hirshfeld surface represents the shortest van der Waals contacts where the π-type halogen bond is located. In addition, the faint red spots indicate separations less than the sum of the van der Waals radii for the C—Cl⋯π contacts. Lastly, dashed lines illustrate the Type I chlorine–chlorine inter­actions observed within (C_6_I_2_Cl_4_)·(nap). This Hirshfeld surface analysis along with the observed bond lengths confirms the ability of C_6_I_2_Cl_4_ to engage in π-type halogen bonds to a polycyclic aromatic hydro­carbon, namely nap.

## 4. Database survey

A search of the Cambridge Crystallographic Database (Version 2023.2.0 Build 3382240; Groom *et al.*, 2016 ▸) using *Conquest* (Bruno *et al.*, 2002 ▸) for structures containing 1,4-di­iodo­perchloro­benzene (C_6_I_2_Cl_4_) in which the I atom is within the van der Waals radius of an aromatic surface revealed only one structure, refcode HONBIY (Bosch, 2019*a* ▸). In particular, this multicomponent solid is a monosolvate of benzene where C_6_I_2_Cl_4_ forms two π-type halogen bonds, generating a similar chevron-like pattern observed in (C_6_I_2_Cl_4_)·(nap).

## 5. Synthesis and crystallization

*Materials and general methods*

The solvent toluene along with the halogen-bond acceptor naphthalene (nap) were both purchased from Sigma-Aldrich Chemical (St. Louis, MO, USA) and used without any additional purification. The halogen-bond donor 1,4-di­iodo­perchloro­benzene (C_6_I_2_Cl_4_) was synthesized utilizing a previously published method (Reddy *et al.*, 2006 ▸).

*Synthesis and crystallization*

The formation of (C_6_I_2_Cl_4_)·(nap) was achieved by dissolving 50.0 mg of C_6_I_2_Cl_4_ in 2.0 mL of toluene and then combined with a 2.0 mL toluene solution containing 13.7 mg of nap (1:1 molar equivalent). Within two days, single crystals suitable for X-ray diffraction were formed upon loss of some of the solvent by slow evaporation.

## 6. Refinement

Crystal data, data collection, and structure refinement details are summarized in Table 1 ▸. Intensity data were corrected for Lorentz, polarization, and background effects using *APEX4* (Bruker, 2021 ▸). Hydrogen atoms bound to carbon atoms were located in the difference Fourier map and were geometrically constrained using the appropriate AFIX commands.

## Supplementary Material

Crystal structure: contains datablock(s) I. DOI: 10.1107/S2056989023008356/jy2037sup1.cif

Structure factors: contains datablock(s) I. DOI: 10.1107/S2056989023008356/jy2037Isup2.hkl

Supporting information file. DOI: 10.1107/S2056989023008356/jy2037Isup3.cml

CCDC reference: 2291675

Additional supporting information:  crystallographic information; 3D view; checkCIF report

## Figures and Tables

**Table 1:** Experimental details

Crystal data	
Chemical formula	C_6_Cl_4_I_2_·C_10_H_8_
*M*_r_	595.82
Crystal system, space group	Triclinic, *P* 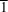
Temperature (K)	100
*a*, *b*, *c* (Å)	5.4830 (7), 6.4533 (11), 12.171 (2)
α, β, γ (°)	87.274 (5), 85.912 (5), 82.629 (9)
*V* (Å^3^)	425.68 (12)
*Z*	1
Radiation type	Mo *K*α
μ (mm^−1^)	4.31
Crystal size (mm)	0.14 × 0.12 × 0.10

Data collection	
Diffractometer	Bruker D8 Venture Duo with Photon III
Absorption correction	Multi-scan (*SADABS*; Krause *et al.*, 2015 ▸)
*T*_min_, *T*_max_	0.626, 0.746
No. of measured, independent and observed [*I* > 2σ(*I*)] reflections	30681, 2518, 2465
*R*_int_	0.050
(sin θ/λ)_max_ (Å^−1^)	0.717

Refinement	
*R*[*F* ^2^ > 2σ(*F* ^2^)], *wR*(*F* ^2^), *S*	0.017, 0.038, 1.09
No. of reflections	2518
No. of parameters	100
H-atom treatment	H-atom parameters constrained
Δρ_max_, Δρ_min_ (e Å^−3^)	0.77, −0.47

Computer programs: *APEX4* (Bruker, 2021 ▸), *SAINT* (Bruker, 2016 ▸), *SHELXT2018/2* (Sheldrick, 2015*a* ▸), *SHELXL* (Sheldrick, 2015*b* ▸), and *OLEX2* (Dolomanov *et al.*, 2009 ▸).

## supplementary crystallographic information

### Crystal data

**Table:**

C_6_Cl_4_I_2_·C_10_H_8_	*Z* = 1
*M**_r_* = 595.82	*F*(000) = 278
Triclinic, *P*1	*D*_x_ = 2.324 Mg m^−^^3^
*a* = 5.4830 (7) Å	Mo *K*α radiation, λ = 0.71073 Å
*b* = 6.4533 (11) Å	Cell parameters from 9944 reflections
*c* = 12.171 (2) Å	θ = 3.2–30.6°
α = 87.274 (5)°	µ = 4.31 mm^−^^1^
β = 85.912 (5)°	*T* = 100 K
γ = 82.629 (9)°	Irregular, clear colourless
*V* = 425.68 (12) Å^3^	0.14 × 0.12 × 0.10 mm

### Data collection

**Table:**

Bruker D8 Venture Duo with Photon III diffractometer	2465 reflections with *I* > 2σ(*I*)
phi and ω scans	*R*_int_ = 0.050
Absorption correction: multi-scan (SADABS; Krause *et al.*, 2015)	θ_max_ = 30.7°, θ_min_ = 3.2°
*T*_min_ = 0.626, *T*_max_ = 0.746	*h* = −7→7
30681 measured reflections	*k* = −9→9
2518 independent reflections	*l* = −17→17

### Refinement

**Table:**

Refinement on *F*^2^	Primary atom site location: dual
Least-squares matrix: full	Hydrogen site location: inferred from neighbouring sites
*R*[*F*^2^ > 2σ(*F*^2^)] = 0.017	H-atom parameters constrained
*wR*(*F*^2^) = 0.038	*w* = 1/[σ^2^(*F*_o_^2^) + 0.4448*P*] where *P* = (*F*_o_^2^ + 2*F*_c_^2^)/3
*S* = 1.09	(Δ/σ)_max_ = 0.001
2518 reflections	Δρ_max_ = 0.77 e Å^−^^3^
100 parameters	Δρ_min_ = −0.47 e Å^−^^3^
0 restraints	

### Special details

**Table:**

Geometry. All esds (except the esd in the dihedral angle between two l.s. planes) are estimated using the full covariance matrix. The cell esds are taken into account individually in the estimation of esds in distances, angles and torsion angles; correlations between esds in cell parameters are only used when they are defined by crystal symmetry. An approximate (isotropic) treatment of cell esds is used for estimating esds involving l.s. planes.
Refinement. A numerical absorption correction was applied based on a Gaussian integration over a multifaceted crystal and followed by a semi-empirical correction for adsorption applied using SADABS (Bruker, 2016). The program SHELXT (Sheldrick, 2015a) was used for the initial structure solution and SHELXL (Sheldrick, 2015b) was used for the refinement of the structure. Both programs were utilized within the OLEX2 software (Dolomanov *et al.*, 2009).

### Fractional atomic coordinates and isotropic or equivalent isotropic displacement parameters (Å^2^)

**Table:**

	*x*	*y*	*z*	*U*_iso_*/*U*_eq_	
I1	0.56547 (2)	0.30084 (2)	0.68774 (2)	0.01552 (4)	
Cl1	0.87192 (8)	0.70258 (6)	0.72721 (3)	0.01816 (8)	
Cl2	0.73398 (7)	0.12959 (6)	0.43367 (3)	0.01748 (8)	
C1	0.8224 (3)	0.4211 (2)	0.57480 (12)	0.0125 (3)	
C2	0.9407 (3)	0.5897 (2)	0.60178 (12)	0.0130 (3)	
C3	0.8824 (3)	0.3322 (2)	0.47217 (12)	0.0127 (3)	
C4	0.5015 (3)	0.0935 (3)	1.02907 (13)	0.0161 (3)	
C5	0.3321 (3)	0.2714 (3)	1.00359 (14)	0.0187 (3)	
H5	0.333552	0.396640	1.041387	0.022*	
C6	0.1657 (3)	0.2649 (3)	0.92489 (15)	0.0214 (3)	
H6	0.053127	0.385222	0.908670	0.026*	
C7	0.1621 (3)	0.0800 (3)	0.86822 (14)	0.0209 (3)	
H7	0.046094	0.076467	0.814172	0.025*	
C8	0.3245 (3)	−0.0955 (3)	0.89020 (14)	0.0189 (3)	
H8	0.320044	−0.218777	0.851101	0.023*	

### Atomic displacement parameters (Å^2^)

**Table:**

	*U*^11^	*U*^22^	*U*^33^	*U*^12^	*U*^13^	*U*^23^
I1	0.01377 (6)	0.01772 (6)	0.01506 (6)	−0.00420 (4)	0.00031 (3)	0.00367 (3)
Cl1	0.02229 (18)	0.01951 (17)	0.01340 (16)	−0.00507 (14)	0.00101 (13)	−0.00473 (13)
Cl2	0.01990 (17)	0.01571 (16)	0.01881 (17)	−0.00845 (13)	−0.00281 (13)	−0.00202 (13)
C1	0.0122 (6)	0.0126 (6)	0.0128 (6)	−0.0027 (5)	−0.0011 (5)	0.0018 (5)
C2	0.0143 (6)	0.0132 (6)	0.0115 (6)	−0.0013 (5)	−0.0023 (5)	−0.0010 (5)
C3	0.0129 (6)	0.0119 (6)	0.0139 (6)	−0.0032 (5)	−0.0032 (5)	0.0007 (5)
C4	0.0150 (7)	0.0205 (7)	0.0129 (6)	−0.0037 (6)	0.0019 (5)	−0.0007 (6)
C5	0.0200 (7)	0.0180 (7)	0.0177 (7)	−0.0022 (6)	0.0023 (6)	−0.0008 (6)
C6	0.0191 (8)	0.0224 (8)	0.0208 (8)	0.0013 (6)	0.0015 (6)	0.0030 (6)
C7	0.0171 (7)	0.0299 (9)	0.0162 (7)	−0.0048 (6)	−0.0015 (6)	−0.0004 (6)
C8	0.0177 (7)	0.0246 (8)	0.0156 (7)	−0.0066 (6)	0.0004 (6)	−0.0035 (6)

### Geometric parameters (Å, º)

**Table:**

I1—C1	2.0929 (15)	C4—C8^ii^	1.419 (2)
Cl1—C2	1.7196 (16)	C5—H5	0.9500
Cl2—C3	1.7252 (16)	C5—C6	1.374 (3)
C1—C2	1.399 (2)	C6—H6	0.9500
C1—C3	1.400 (2)	C6—C7	1.410 (3)
C2—C3^i^	1.401 (2)	C7—H7	0.9500
C4—C4^ii^	1.430 (3)	C7—C8	1.376 (3)
C4—C5	1.418 (2)	C8—H8	0.9500
			
C2—C1—I1	120.33 (11)	C4—C5—H5	119.6
C2—C1—C3	118.93 (13)	C6—C5—C4	120.84 (16)
C3—C1—I1	120.74 (11)	C6—C5—H5	119.6
C1—C2—Cl1	120.19 (12)	C5—C6—H6	120.0
C1—C2—C3^i^	120.74 (14)	C5—C6—C7	120.05 (16)
C3^i^—C2—Cl1	119.07 (11)	C7—C6—H6	120.0
C1—C3—Cl2	120.49 (12)	C6—C7—H7	119.6
C1—C3—C2^i^	120.33 (13)	C8—C7—C6	120.82 (16)
C2^i^—C3—Cl2	119.16 (11)	C8—C7—H7	119.6
C5—C4—C4^ii^	119.00 (19)	C4^ii^—C8—H8	119.8
C5—C4—C8^ii^	122.12 (15)	C7—C8—C4^ii^	120.41 (16)
C8^ii^—C4—C4^ii^	118.88 (19)	C7—C8—H8	119.8
			
I1—C1—C2—Cl1	−1.15 (18)	C3—C1—C2—C3^i^	−0.4 (2)
I1—C1—C2—C3^i^	178.35 (11)	C4^ii^—C4—C5—C6	0.5 (3)
I1—C1—C3—Cl2	3.33 (18)	C4—C5—C6—C7	−0.1 (3)
I1—C1—C3—C2^i^	−178.35 (11)	C5—C6—C7—C8	−0.3 (3)
C2—C1—C3—Cl2	−177.92 (11)	C6—C7—C8—C4^ii^	0.2 (3)
C2—C1—C3—C2^i^	0.4 (2)	C8^ii^—C4—C5—C6	179.92 (16)
C3—C1—C2—Cl1	−179.91 (11)		

Symmetry codes: (i) −*x*+2, −*y*+1, −*z*+1; (ii) −*x*+1, −*y*, −*z*+2.
